# New Clues to Cardiovascular Disease: Erythrocyte Lifespan

**DOI:** 10.14336/AD.2023.0506

**Published:** 2023-12-01

**Authors:** Ziyu Lu, Yuanmin Li

**Affiliations:** Department of Cardiology, the Second Affiliated Hospital, Shandong First Medical University & Shandong Academy of Medical Sciences, Taian, China

**Keywords:** erythrocyte lifespan, heart failure, atherosclerosis, hypertension, statins, heart chamber assist device

## Abstract

Determination of erythrocyte lifespan is an important part of the diagnosis of hemolytic diseases. Recent studies have revealed alterations in erythrocyte lifespan among patients with various cardiovascular diseases, including atherosclerotic coronary heart disease, hypertension, and heart failure. This review summarizes the progress of research on erythrocyte lifespan in cardiovascular diseases.

## Introduction

The lifespan of erythrocytes is the duration they remain in the bloodstream. In healthy individuals, erythrocytes usually survive for an average of 120 days [1]. However, previous research has demonstrated that several conditions, including hereditary xerocytosis (HX), severe aplastic anemia (SAA), and sickle cell anemia (SCA), may result in a shortened lifespan of erythrocytes, leading to anemia [2-7]. Patients with renal insufficiency may experience varying degrees of anemia [8, 9] that could be linked to reduced production of endogenous erythropoietin (EPO) and iron deficiency [10, 11]. Furthermore, shortened erythrocyte lifespan has been observed in patients on dialysis with renal insufficiency and proteinuria [12-18], as well as in those with diabetes [19-25].

Anemia, which can result from a shortened lifespan of erythrocytes, is closely associated with cardiovascular diseases (CVD) [26] and increases the risk of developing CVD [27]. Recently, more attention has been given to the alterations in erythrocytes' lifespan associated with cardiovascular diseases such as heart failure, hypertension, and atherosclerosis (AS). Additionally, statins, commonly used to treat CVD, have been linked to a shortened lifespan of erythrocytes as a side effect.

This review aims to summarize the changes in erythrocyte lifespan in CVD patients and suggest future research directions. Our goal is to highlight the importance of the erythrocyte lifespan for researchers.

## Role of Erythrocyte lifespan in cardiovascular diseases

Erythrocytes are unique in lacking a nucleus and having a double concave disc shape. They possess several physiological characteristics, including plasticity, suspension stability, and osmotic fragility. Erythrocytes are primarily responsible for transporting oxygen and carbon dioxide [28] and play a crucial role in buffering acid-base substances in the blood [29], clearing immune complexes [30], metabolizing systemic nitric oxide, regulating redox reactions, and modulating blood viscosity [31]. Erythrocyte production is mainly influenced by EPO and sex hormones. Besides, thyroid, adrenocortical, and growth hormones can indirectly impact erythropoiesis.

As the most abundant cell type in circulation, alterations in erythrocyte lifespan can contribute to the development of cardiovascular diseases such as acute heart failure, hypertension, and AS.

### Erythrocyte lifespan and acute heart failure

Patients with heart failure have a higher risk of developing anemia [32, 33] due to excessive eryptosis, a process characterized by erythrocyte contraction, phosphatidylserine (PS) exposure, and clustering of band-3 on eryptotic erythrocytes, along with a decrease in CD47 levels [34-36]. Mahmud *et al.* [37] found that erythrocyte PS exposure was more prominent in rats with acute heart failure, leading to increased phagocytosis by macrophages and a shortened erythrocyte lifespan. The authors analyzed erythrocytes from five patients with acute heart failure and found a significant increase in PS binding to annexin V, indicating erythrocyte senescence [38]. These findings suggest that the erythrocyte death rate is accelerated, and the erythrocyte lifespan is shortened in rodents and human patients with heart failure. Oxidative stress is another factor that affects erythrocyte lifespan [39], and Mahmud *et al*. [37] suggested that sustained oxidative stress in patients with acute heart failure contributes to accelerated eryptosis.

Attanasio *et al*. [40] conducted a study on 22 acute heart failure patients and 10 healthy individuals as the control group, all with the same number of erythrocytes. The rate of eryptosis was measured using annexin V, and they found that the binding rate of PS in erythrocytes to annexin V was significantly higher in the patient group than in the control group. The study results indicate that the rate of eryptosis increased in patients with acute heart failure, leading to anemia and a shortened erythrocyte lifespan. Furthermore, the findings suggest that oxidative stress can accelerate the rate of eryptosis [40].

### Erythrocytes lifespan and hypertension

Hypertension is China's most prevalent chronic non-communicable disease, with 244.5 million patients in 2015 [41]. Hyperlipidemia often accompanies hypertension in diabetic patients [42]. Pinzón-Díaz *et al*. [43] investigated erythrocyte lifespan in patients with hypertension and hyperlipidemia. They classified 81 patients as healthy (NT), hypertensive (HT), hyper-lipidemic (ND), and hypertensive and hyperlipidemic (HTD). Patients with HTD had the highest calcium ion levels in erythrocytes, followed by those with HT. Regardless of hyperlipidemia, calcium ions in erythrocytes were elevated in patients with hypertension, leading to PS exposure. All three groups (HT, ND, and HTD) had lower glutathione (GSH) concentrations than the NT group. Both HT and HTD groups had a higher degree of lipid peroxidation. Pinzón-Díaz *et al*. [43] suggested that increased intracellular calcium concentration in erythrocytes in patients with hypertension can lead to PS exposure, but it is not entirely proportional. Because an increase in blood cholesterol causes an increase in cholesterol on the cell membrane [44], the enzymes required for PS inversion could weaken the sensitivity to calcium ions, decreasing the activity of inverting enzymes. At the same time, the decrease of GSH on the erythrocyte membrane could also weaken the antioxidant capacity of erythrocytes and shorten their lifespan.

Pinzón-Díaz *et al*. observed that hypertension increased eryptosis due to oxidative stress, although other molecular mechanisms may be at play. Huang *et al*. [45] created a hypertension mice model and injected angiotensin II (Ang II) in the experimental group and saline in the control group. Their study showed that Ang II decreased the expression of CD47 on erythrocyte surfaces and increased the binding of Annexin V to PS. However, losartan, an angiotensin 1 receptor antagonist, reversed these effects. Angiotensin II also reduced antioxidant enzymes, and the addition of antioxidant N-acetylcysteine (NAC) produced similar effects to losartan and maintained a redox balance. The authors suggested that angiotensin II induces erythrocyte redox imbalance through the angiotensin II type 1 receptor. Pinzón-Díaz *et al*. also proposed that angiotensin II may promote the formation of NADPH oxidase and reactive oxygen species (ROS), which generate superoxide ions in the cells. Interestingly, Guimarães-Nobre *et al*. [46] demonstrated that in sickle cell anemia patients, moderate concentrations of angiotensin II solution reduced the binding of PS to Annexin V through angiotensin 1 receptor (ATR1), possibly by reducing PS valgus. However, high and low concentrations of Ang II could not reduce PS valgus, suggesting that angiotensin II and ATR1 have different effects on erythrocytes in different diseases.

### Erythrocyte lifespan and AS

Recent studies have indicated that erythrocyte lifespan may play a crucial role in the progression of AS [47]. Individuals with AS were reported to have erythrocytes with a reduced lifespan, making them more susceptible to spleen-mediated removal from circulation [47]. Delbosc *et al*. [48] conducted a study on rabbits and induced AS through a high-fat diet. The study found that hypercholesterolemic rabbits had erythrocytes with accelerated senescence and shortened lifespan. During the early stages of AS, erythrocytes were detected in the vessel wall of arteries and were phagocytosed by vascular smooth muscle cells. Both humans and rabbits with early-stage AS exhibited iron and Hb deposits in their arteries, which promoted the progression of AS. The hemolysis of erythrocytes in AS was caused by oxidized low-density lipoprotein (ox-LDL) and lipids, resulting in a shorter erythrocyte lifespan and increased iron and heme production, which further promoted the oxidation of lipids and the production of atheromatous substances and endothelial stress [49]. Hemeoxygenase-1 (HO-1) has been shown to have anti-AS effects[50], and its production is increased in AS. Additionally, Sánchez *et al*. [51] observed increased erythropoiesis in mice with AS.

Wang *et al*. [52] demonstrated that AS was associated with Jak2 gene mutation. In their study, they transplanted bone marrow cells from Jak2V617F, a prevalent mutation that causes myeloproliferative disorders [53], and wild-type (WT) mice into low-density lipoprotein receptor knockout mice and fed them a high-fat diet. Their results demonstrated that the Jak2V617F mutation promoted the occurrence and development of AS. Individuals carrying the Jak2V617F mutation had increased erythrocyte numbers and decreased CD47 expression on erythrocytes, which promoted erythrocyte phagocytosis and shortened the lifespan of erythrocytes. The decrease in CD47 on the surface of erythrocytes led to erythrocyte phagocytosis, which shortened the lifespan of erythrocytes. Jak2V617F mice had reduced levels of MerTK, a receptor expressed on macrophages that promotes phagocytosis, while erythrocyte phagocytosis inhibited efferocytosis. Efferocytosis can prevent secondary necrosis of near-death cells, thereby preventing the release of inflammatory substances. The Jak2V617F mutation can promote thrombocytosis, enhance platelet activity, and further promote the occurrence of AS.

### Erythrocyte lifespan and thrombus

Red blood cells play a role in the formation of thrombi, which can lead to heart attacks. Patients with Sickle Cell Anemia (SCA) are at a significantly higher risk of pulmonary embolism than healthy individuals [54-58]. These emboli form within the lungs rather than from lower limb deep veins [55, 56]. Patients with hereditary spherocytosis (HS) also have an increased risk of arterial thrombosis after splenectomy [59]. Deformed erythrocytes can increase blood viscosity [60] and promote thrombosis in patients with SCA and HS [61]. Damaged erythrocytes can release Hemoglobin (Hb) and Adenosine Diphosphate (ADP), which can promote platelet aggregation and activation [62-64]. PS exposure in erythrocytes drives thrombin production [65-69]. However, it did not predict thrombotic risk in HS mice [70]. It was reported that PS exposure increases in erythrocytes [70-72], and erythrocyte lifespan is shortened in patients with SCA. Elevated prothrombin fragment 1.2 has been observed in patients with SCA, and it was associated with PS in erythrocytes [73]. Furthermore, erythrocytes may increase thrombin production and inhibit thrombolysis [61].

In a study by Schleicher *et al*. [74], platelets were found to express FasL, a ligand for cell death receptors, which induced apoptosis. Similarly, erythrocytes were also found to express FasR, a cell death receptor [75]. Klatt *et al*. [76] discovered that erythrocytes can expose FasL on platelets, leading to FasR activation in erythrocytes. This binding induced PS exposure on erythrocytes and promoted platelet activation and thrombosis. Additionally, erythrocytes can contribute to thrombosis by increasing blood viscosity [77, 78]. Dayal *et al*. [79] demonstrated that mice deficient in superoxide dismutase-1 had a shorter erythrocyte lifespan, exhibited eryptosis, and experienced faster thrombosis, indicating that superoxide dismutase is crucial for erythrocyte lifespan and thrombosis [80].

## Mechanisms linking erythrocyte lifespan and cardiovascular diseases

### Oxidative stress and inflammation

Patients with heart failure experience accelerated eryptosis, the process of programmed cell death in erythrocytes. This is caused by oxidative stress or activation of calcium-sensitive potassium channels by calcium ions, leading to erythrocyte shrinkage and increased exposure of PS on erythrocytes. As a result, the lifespan of erythrocytes is shortened in these patients [81].

**Figure 1.**
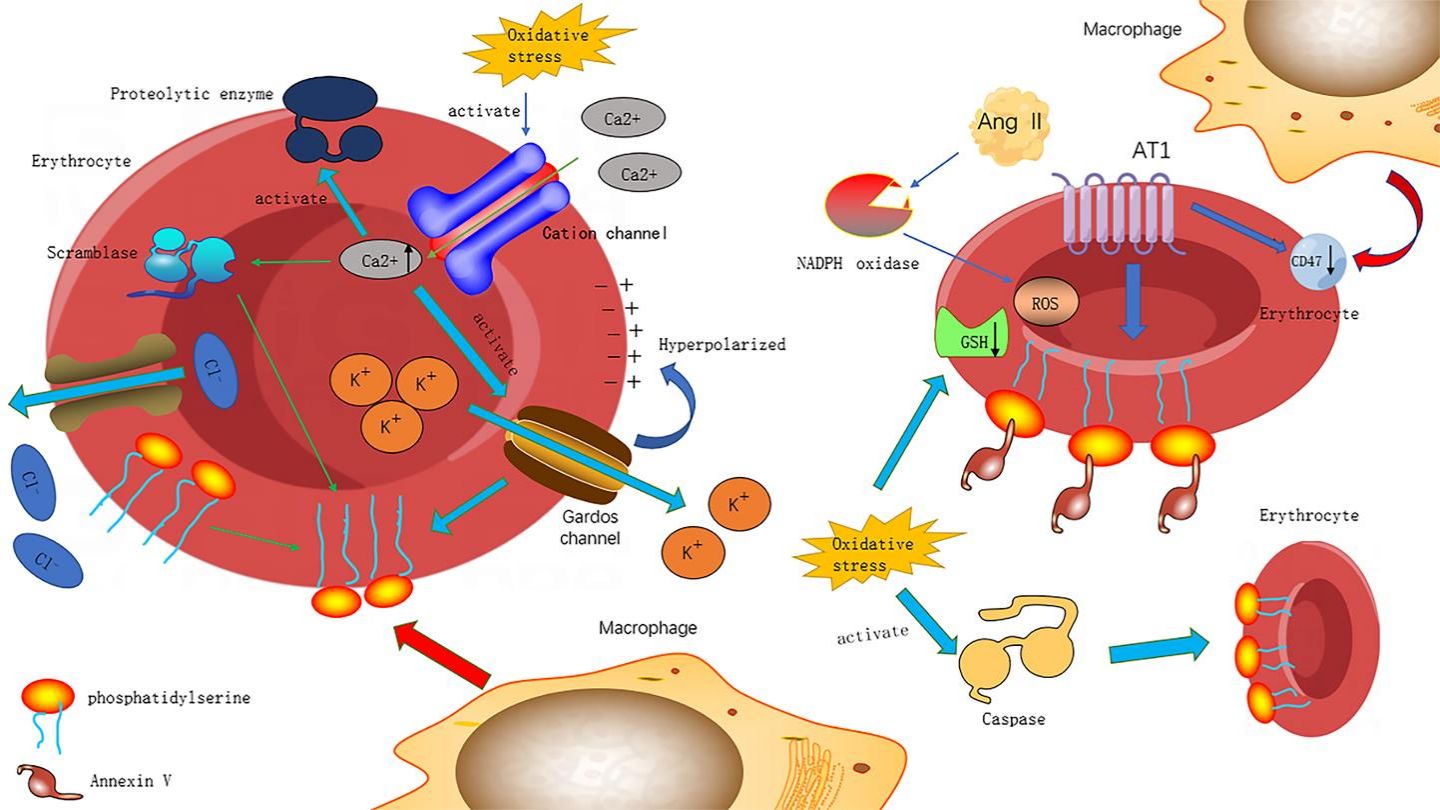
**The potential mechanism of oxidative stress leading to shortened lifespan of erythrocyte**. Oxidative stress triggers the activation of caspase, an enzyme that stimulates the exposure of phosphatidylserine (PS) on the erythrocyte membrane. Additionally, oxidative stress activates cation channels on the erythrocyte surface, leading to an influx of calcium ions into the cell, which enhances calcium activity. The increased concentration and activity of calcium ions in erythrocytes stimulates scramblase, which transfers PS from the inside to the outside of the erythrocyte. Intracellular calcium also activates calcium-sensitive potassium channels, known as Gardos channels. This leads to an increase in potassium ion outflow and membrane hyperpolarization, driving anion outflow through chloride channels. As a result, there is a loss of intracellular potassium chloride, a decrease in intracellular osmotic pressure, and erythrocyte contraction. The activation of Gardos channels also promotes PS exposure. Intracellular calcium ions can activate proteolytic enzymes, leading to the degradation of erythrocyte cytoskeleton. Moreover, angiotensin II reduces the expression of CD47 on the surface of erythrocytes but increases the binding of Annexin V to PS. It promotes the formation of NADPH oxidase and reactive oxygen species (ROS), leading to the oxidation of cells. During oxidative stress, the level of glutathione (GSH) on erythrocytes decreases, and the antioxidant capacity of erythrocytes decreases.

As shown in Figure 1, oxidative stress induces PS exposure through several mechanisms. Firstly, it activates caspase, an apoptosis-related enzyme expressed in erythrocytes, leading to PS exposure on the erythrocyte membrane [37, 82]. Secondly, oxidative stress activates cation channels on the erythrocyte surface, resulting in calcium influx into erythrocytes and increased calcium activity. This high calcium concentration in erythrocytes stimulates scramblase, which transfers PS from inside to outside erythrocytes [37, 81, 82]. Additionally, the activation of Gardos channels, which are calcium-sensitive potassium channels, promotes PS exposure through increased potassium ion outflow, membrane hyperpolarization, and anion outflow through chloride channels. These processes lead to the loss of intracellular potassium chloride, decreased intracellular osmotic pressure, and erythrocyte contraction [81]. Intracellular calcium ions could also activate proteolytic enzymes, causing the degradation of the erythrocyte cytoskeleton [37, 82]. Energy depletion during heart failure damages the supply of GSH and weakens the antioxidant barrier of erythrocytes, leading to eryptosis [37, 82]. This process can be activated through the hyperosmotic shock pathway, where cationic channels are stimulated by the release of prostaglandin E2 [83]. Furthermore, sepsis caused by excessive inflammation and tissue damage can promote eryptosis by increasing the production of ceramide, which facilitates the entry of calcium ions into erythrocytes [84].

Inflammation can have a negative impact on erythrocyte deformability. Normally, erythrocytes can undergo plastic deformation and regain their biconcave disc shape after passing through capillaries. However, erythrocytes lose this ability in the presence of inflammation and cannot maintain their biconcave disc shape after deformation [85]. Hyperactivated platelets and increased adhesion to erythrocytes may also impair erythrocyte deformability [85]. Furthermore, when erythrocytes interact with inflammatory factors, it can lead to pathological deformation and, ultimately, eryptosis [85].

### Anemia and reduced oxygen delivery

Anemia triggers the sympathetic nerve and activates RAAS, causing an increase in oxidative stress [31]. Oxidative stress can damage the erythrocyte membrane, reducing erythrocyte lifespan [86]. Additionally, anemia could exacerbate coronary heart disease by causing tissue hypoxia, increasing anemic patients' heart rate. This acceleration, coupled with increased preload, could lead to left ventricular hypertrophy and cardiac cavity enlargement [87], ultimately resulting in heart failure. Anemia is also closely associated with hypertension, arrhythmia, and cerebral stroke [86].

### Iron and pro-inflammatory molecule release

Approximately two-thirds of the body's iron is found in erythrocytes [88], which is vital to their lifespan [89]. Iron deficiency can lead to increased programmed cell death, shortened erythrocyte lifespan, and anemia [90]. A shortage of iron results in more calcium ions entering the red blood cells, which is the leading cause of their shortened lifespan [90]. In conditions like thalassemia, iron stored outside red blood cells leads to non-transferrin-plasma iron (NTPI) accumulation, which is toxic and can cause lipid peroxidation on cell membranes, damaging cells and causing hemosiderin cardiomyopathy [91].

Ferroptosis is a type of non-apoptotic cell death caused by the RAS-selective lethal small molecule erastin and is different from other forms of iron metabolism. During ferroptosis, the cystine/glutamate antiporter is inhibited [92], leading to a depletion of GSH, an increase in oxidative stress, and the inhibition of glutathione peroxidase 4 (GPX4) [93]. These effects increase the peroxidation of lipids and can destroy cell membranes, including those of red blood cells [94].

The destruction of erythrocytes leads to the release of various components, which induces aseptic inflammation via Toll-like receptors (TLR) and NOD-like receptors (NLR) [95]. Heme can cause oxidation and inflammation [96] and activate TLR4 to induce macrophages to secrete TNF-α [97]. Additionally, heme can activate neutrophils and enhance the production of ROS and interleukin-8 (IL-8) [98]. The prolonged state of heme overload can result in heart damage [99]. Studies have shown that methemoglobin can cause extensive neuroinflammation through TLR4 after subarachnoid hemorrhage [100]. Upon stimulation, activated NOD-like receptors (NLR) can promote the activation of caspase-1, which converts the IL-1 family of cytokines into their active forms, IL-1β and IL-18, leading to cell death [101].

Cardiovascular diseases, including acute heart failure, hypertension, thrombosis, and AS, have been linked to the shortened lifespan of erythrocytes. However, the exact nature of the relationship between erythrocyte lifespan and cardiovascular disease is still being explored.

## Factors affecting the lifespan of erythrocytes

The lifespan of erythrocytes is influenced by various factors, including genetics, cardiovascular diseases, and environmental factors. HX, an inherited congenital hemolytic disease, is one example of how genetics can play a crucial role. HX pathogenesis is believed to be induced mainly by a PIEZO1 mutation, which leads to calcium influx, Gardos channel activation, and potassium and water outflow [2, 4]. As a result, an imbalance of sodium and potassium ions in erythrocytes occurs, leading to erythrocyte dehydration, decreased deformability, and shortened lifespan [2, 4]. Smoking is another factor that significantly affects erythrocyte lifespan. Studies have shown that smoking reduces CD47 expression on erythrocyte surfaces, leading to shortened erythrocyte lifespan [102]. Moreover, diabetes, associated with widespread oxidative stress and inflammation, has been linked to shortened erythrocyte lifespan in several studies [19-25]. Patients with renal insufficiency and proteinuria undergoing dialysis have also shown shortened erythrocyte lifespan [12-18]. Finally, some iatrogenic factors also affect the lifespan of erythrocytes.

### Statins

Due to their plaque-stabilizing and lipid-lowering effects, statins were a significant breakthrough in the treatment of various diseases, including hyperlipidemia [103], hypercholesterolemia [104], AS, and leukemia [52, 105]. Biswas *et al*. [106, 107] found that arsenic reduced erythrocyte lifespan, which was restored after treatment with atorvastatin and N-acetylcysteine (NAC). Studies have shown that atorvastatin can reverse the altered properties of erythrocyte membranes in patients with hypercholesterolemia [108]. Similarly, simvastatin was reported to decrease lipid peroxidation of erythrocyte membranes [109]. Lipid-lowering therapy with atorvastatin combined with ezetimibe or atorvastatin alone has also been shown to improve erythrocyte membrane parameters in patients with coronary artery disease [110].

However, there are conflicting findings about the effects of statins on erythrocyte lifespan. *In vitro* experiments by Rana *et al*. [80] showed that atorvastatin administration at different concentrations (1-10 µM) induced oxidative stress in erythrocytes, resulting in decreased activities of GSH peroxidase, superoxide dismutase, and catalase, and shortened erythrocyte lifespan. Al Mamun Bhuyan *et al*. [111] showed that erythrocytes exposed to a 1 μg/ml solution of simvastatin experienced eryptosis, characterized by an increase in the percentage of PS exposure and induction of calcium ions into the erythrocytes, causing oxidative stress and eryptosis.

Therefore, more studies are necessary to fully understand the effect of statins on erythrocyte lifespan, given the current ambiguity on this matter.

### The cardiac chamber assists devices

Advancements in science and technology have led to the development of intra-cardiac assist devices, which can now be used to treat end-stage heart failure and valvular heart disease. The left ventricular assist device (LVAD) is one such device that is used to slow down the pressure of ventricular pumping by introducing blood from the ventricle into the device, which is then pumped into the aorta. This device is commonly used in patients with end-stage heart failure.

Taimeh *et al*. [112] investigated the relationship between erythrocyte lifespan and the constant-flow left ventricular assist device (CF-LVAD). The study showed that erythrocytes lifespan in patients with CF-LVAD was shorter than that of normal subjects. Additionally, patients with thrombus formation had a shorter erythrocyte lifespan than those without thrombus, and both groups experienced anemia. The study suggested that mechanical damage to erythrocytes by the pump is the primary mechanism of anemia [112]. Although CF-LVAD can improve survival and alleviate symptoms in patients with end-stage heart failure, it comes with complications, such as anemia and infections [113].

Vrtovec *et al*. [114] found that non-anemic patients had twice the survival rate of anemic patients after 6 months of LVAD application. Infection is also a common complication in patients with LVAD [115], and Hernandez *et al*. [116] suggested that readmission rates could be reduced by anticoagulation and treatment of complications. Therefore, it is essential to give adequate attention to the potential complications of CF-LVAD, including the shortened lifespan of erythrocytes, in the treatment of patients with end-stage heart failure.

## Erythrocyte lifespan assays

### Discriminative agglutination method

In 1919, Ashby devised the discriminative agglutination method to determine erythrocyte lifespan. This method involved injecting type O blood erythrocytes into individuals with type A blood, followed by mixing blood samples with anti-A serum after transfusion to produce an agglutination reaction. The survival time for type O erythrocytes could be tracked by collecting blood regularly for agglutination reaction [117-121]. Nonetheless, implementing this method in a clinical setting proved challenging.

### Labeling method

The 51Cr and 15N glycine labeling methods were developed later for measuring erythrocyte lifespan. 51Cr can label erythrocytes of all ages, and when hexavalent chromium ions enter the erythrocytes, they are reduced to trivalent chromium ions and remain in the erythrocyte until the cell dies. This method involves injecting the labeled erythrocytes into subjects and drawing blood regularly to measure the radiation intensity of 51Cr. As time passes, the labeled erythrocytes gradually die out, and the radioactivity of 51Cr decreases. The survival curve of erythrocytes can be drawn based on the decreasing rate of radiation intensity, and the lifespan of erythrocytes can be determined. However, this method is time-consuming, and the results are not the exact value of erythrocyte life [122]. Correction is also required using a coefficient table. Additionally, this method is not suitable for children or pregnant women due to the use of radioactive elements.

### Creatine method

Kameyama *et al*. [123] proposed a method for determining erythrocyte lifespan based on the logarithm of creatine through a linear model. The formula is logeEC=-0.04379MRBC+2.882, and the transformed formula is MRBC=-22.84logeEC+65.83, where EC represents erythrocyte creatine (μmol/g Hb), and MRBC represents mean erythrocyte age (days). However, this method has a complex formula and is not straightforward to obtain results. Moreover, it is significantly influenced by renal function.

### CO expiratory method

The carbon monoxide (CO) breath test is a commonly used method to measure erythrocyte lifespan. When eryptosis occurs, endogenous CO is produced by heme oxidation, which accounts for approximately 70% of the total endogenous CO produced by the body. These CO are eliminated through the lungs [124, 125]. By measuring the concentration of CO in the environment, the concentration of endogenous CO can be determined, and the metabolic rate of erythrocytes can be calculated. When the rate of hemoglobin synthesis equals the rate of decomposition, a dynamic balance is achieved, and the average lifespan of erythrocytes on that day is equal to the total amount of hemoglobin/hemoglobin decomposition. One millimole of hemoglobin contains four millimoles of heme, and one millimole of heme oxidizes to produce one millimole of CO. Thus, the above formula can be modified to the total amount of heme-derived CO/CO lung output on the same day, and the formula is erythrocyte lifespan = (4 × Hb × 22,400)/(0.7 × endPCO × 64,400 × 1,440) × (Vb/Vt) [Hb: hemoglobin (g/L); 22,400: standard state gas molecular volume (mL); 4: 1 mmol hemoglobin releases 4 mmol CO; 0.7: ratio of CO produced by heme to endogenous CO; 64,400: blood red egg white fraction; 1,440: total minutes in a day (min); endPCO: endogenous CO partial pressure (ppm); Vb: blood volume (mL); Vt: resting alveolar ventilation (mL/min)]. The values of Vb and Vt are approximately the same. Thus, erythrocyte lifespan = (Hb × K)/endPCO [K: 1,380, unit: mL/(dg)] [124-127]. Hemoglobin concentration can be determined through a routine blood test, while the endogenous CO concentration during expiration can be measured using specific instruments. As a result, the CO breath test is relatively simple and can be conducted in a clinical setting.

However, due to the requirement for a greater amount of sample gas than that exhaled by a healthy individual, at least two collections are necessary to obtain the required amount of gas, which may result in errors. In the case of patients with chronic obstructive pulmonary disease (COPD), exhalation is difficult, and the error may be magnified. Therefore, research on how to reduce this error is needed.

## Clinical significance.

### The potentials of erythrocyte lifespan measurement in clinical diagnosis and prognosis

The measurement of erythrocyte lifespan plays a crucial role in clinical practice, serving as the "gold standard" for the diagnosis of hemolytic diseases and aiding in the investigation of therapeutic effects and mechanisms of such diseases. For example, the erythrocyte lifespan measurement can detect hemolysis and guide compensatory bone marrow hematopoiesis in patients with heart valve implantation. Additionally, it can reveal the effects of exercise on erythrocytes and the causes of secondary polycythemia at high altitudes [128].

Traditionally, a shortened erythrocyte lifespan was associated with hemolytic diseases in the blood system, but now it also suggests the possibility of other system diseases, including cardiovascular, endocrine, urinary, and others.

Large-scale population studies are necessary to confirm the diagnostic value of erythrocyte lifespan in cardiovascular diseases, particularly in risk stratification and prognosis evaluation.

### Potential therapeutic strategies targeting erythrocyte lifespan and future research directions

One potential therapeutic strategy for addressing shortened erythrocyte lifespan is to target risk factors such as high blood pressure, blood sugar, and lipids. Additionally, treatments like enhanced external counterpulsation (EECP) and cardiac rehabilitation may help improve erythrocyte lifespan. Hyperbaric oxygen is also being studied for its potential benefits on erythrocyte lifespan.

Dapagliflozin is a drug that can promote erythropoietin (EPO) production [129], which has been shown to protect erythrocytes from oxidative stress [130, 131]. EPO has been found to reduce the area of myocardial infarction in animal experiments during myocardial ischemia-reperfusion [132]. However, according to Gao *et al*. [133], EPO does not improve cardiac function in patients with acute myocardial infarction. Nevertheless, EPO certainly benefits erythrocytes by preventing oxidative stress, though further studies are needed to determine if it affects erythrocyte lifespan.

Nitric oxide (NO) is a signal molecule that can enter erythrocytes and inhibit oxidative stress [5, 89]. Erythrocytes also contain nuclear factor (NFκB), which prevents eryptosis [134] and may be related to erythrocyte aging [135]. NFκB is almost absent in old erythrocytes, which could be a potential future research direction for erythrocyte lifespan.

Other areas of research that merit investigation include the effect of chronic heart failure on erythrocyte lifespan, the use of statins in patients with hyperlipidemia and anemia, and ways to improve erythrocyte lifespan in patients with CF-LVAD and artificial valves. Chinese herbal medicines are also being used to treat heart failure in China, and it remains to be seen whether they can prolong erythrocyte lifespan when combined with current treatment protocols.

## Conclusion

This paper aimed to shed light on the relationship between erythrocyte lifespan and cardiovascular diseases. The shortening of erythrocyte lifespan in patients with cardiovascular diseases is linked to oxidative stress, increased calcium ion concentration, decreased activity of GSH peroxidase and Superoxide dismutase (SOD), and increased phosphatidylserine exposure. However, this study has limitations due to the lack of research on erythrocyte lifespan. Future research can focus on 1) investigating the effect of other diseases on erythrocyte lifespan, 2) exploring the impact of different drugs on erythrocyte lifespan, 3) identifying additional molecular mechanisms that contribute to the shortening of erythrocyte lifespan, and 4) investigating the relationship between erythrocyte lifespan and aging to determine whether prolonging erythrocyte lifespan can delay aging.
